# Supplementary material for: Optimization of differentiation and transcriptomic profile of THP-1 cells into macrophage by PMA
Source: PLoS One. 2023 Jul 17;18(7):e0286056. doi: 10.1371/journal.pone.0286056 (PMC10351730; doi:10.1371/journal.pone.0286056)
Supplement: S1 File — (ZIP) [file pone.0286056.s001.zip › supporting files/RNA-seq report.zip!/RNA-seq report/5_KEGGEnrichment_result/induce_vs_control/Enrich_plot/induce_vs_control.DEG_down_KEGG_enriched.pdf]

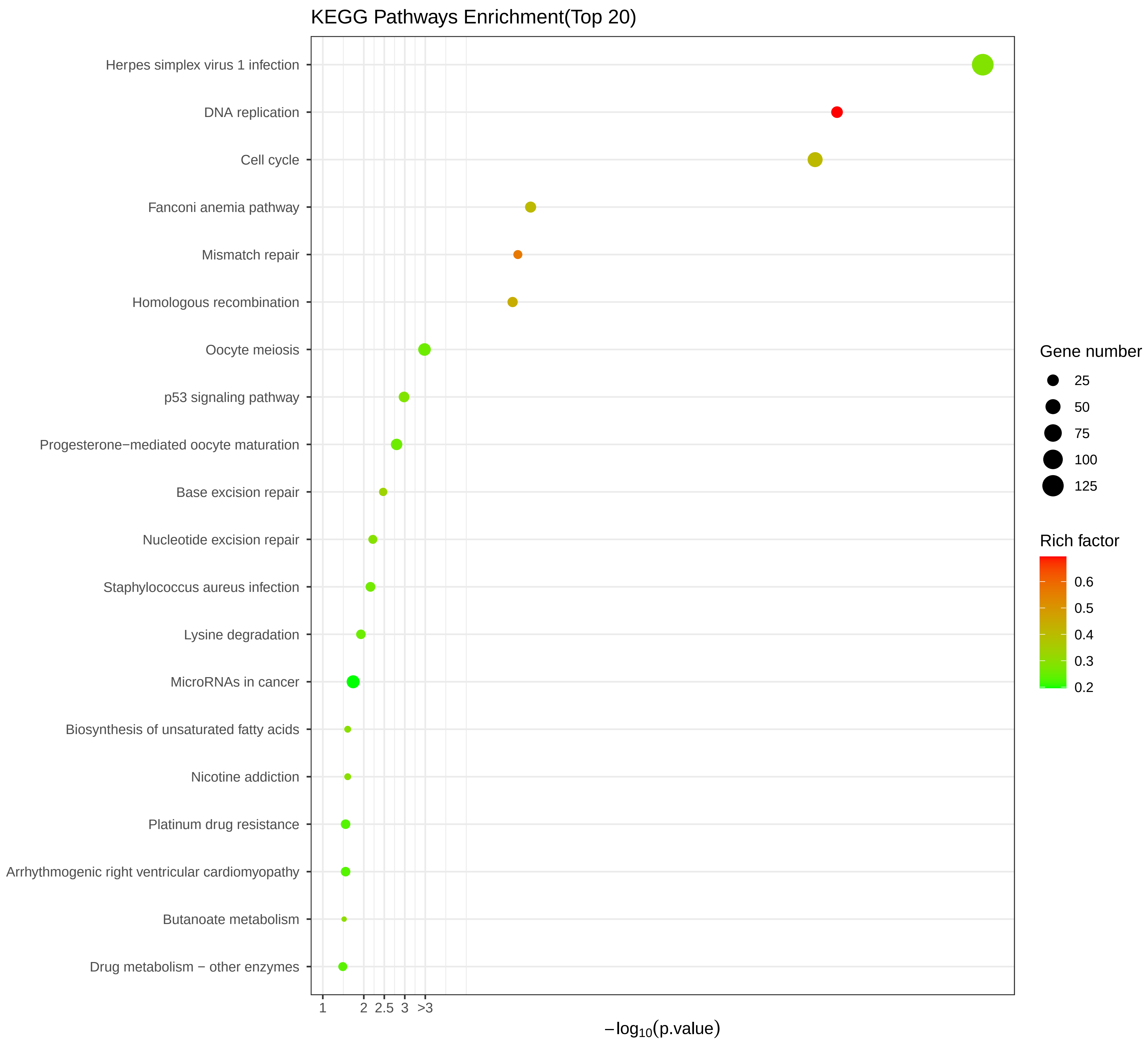

KEGG Pathways Enrichment(Top 20)
Herpes simplex virus 1 infection
DNA replication
Cell cycle
Fanconi anemia pathway
Mismatch repair
Homologous recombination
Oocyte meiosis
p53 signaling pathway
Progesterone−mediated oocyte maturation
Base excision repair
Nucleotide excision repair
Staphylococcus aureus infection
Lysine degradation
MicroRNAs in cancer
Biosynthesis of unsaturated fatty acids
Nicotine addiction
Platinum drug resistance
Arrhythmogenic right ventricular cardiomyopathy
Butanoate metabolism
Drug metabolism − other enzymes
1
2
2.5
3
>3
$-\log_{10}(\text{p.value})$
Gene number
25
50
75
100
125
Rich factor
0.6
0.5
0.4
0.3
0.2
